# Hemidesmosome-Related Keratin Filament Bundling and Nucleation

**DOI:** 10.3390/ijms22042130

**Published:** 2021-02-21

**Authors:** Marcin Moch, Rudolf E. Leube

**Affiliations:** Institute of Molecular and Cellular Anatomy, Rheinisch-Westfälische Technische Hochschule Aachen, 52062 Aachen, Germany; mmoch@ukaachen.de

**Keywords:** keratin intermediate filament, hemidesmosome, focal adhesion, actin, microtubule, integrin beta 4, integrin beta 5, BPAG-1, paxillin, talin

## Abstract

The epithelial cytoskeleton encompasses actin filaments, microtubules, and keratin intermediate filaments. They are interconnected and attached to the extracellular matrix via focal adhesions and hemidesmosomes. To study their interplay, we inhibited actin and tubulin polymerization in the human keratinocyte cell line HaCaT by latrunculin B and nocodazole, respectively. Using immunocytochemistry and time-lapse imaging of living cells, we found that inhibition of actin and tubulin polymerization alone or in combination induced keratin network re-organization albeit differently in each situation. Keratin filament network retraction towards the nucleus and formation of bundled and radial keratin filaments was most pronounced in latrunculin-B treated cells but less in doubly-treated cells and not detectable in the presence of nocodazole alone. Hemidesmosomal keratin filament anchorage was maintained in each instance, whereas focal adhesions were disassembled in the absence of actin filaments. Simultaneous inhibition of actin and tubulin polymerization, therefore, allowed us to dissect hemidesmosome-specific functions for keratin network properties. These included not only anchorage of keratin filament bundles but also nucleation of keratin filaments, which was also observed in migrating cells. The findings highlight the fundamental role of hemidesmosomal adhesion for keratin network formation and organization independent of other cytoskeletal filaments pointing to a unique mechanobiological function.

## 1. Introduction

The keratin cytoskeleton is a hallmark feature of epithelial cells [1,2]. It consists of a filamentous cytoplasmic network with unique biomechanical properties that is connected to desmosomes at cell-cell adhesion sites and to hemidesmosomes at epithelial-extracellular matrix (ECM) interfaces [3,4,5,6]. Its 3D organization and dynamic features rely on the other two major cytoskeletal filament systems, i.e., the actin-based microfilaments, which are anchored to adherens junctions at cell-cell borders and to focal adhesions at cell-ECM contacts, and the tubulin-based microtubules [7,8,9,10,11]. The resulting highly complex transcellular scaffold supports epithelial tissue cohesiveness and adhesion. Intense research efforts are directed towards elucidation of the specific contribution of the different filament-adhesion systems to epithelial function and homeostasis. The current study focuses on keratin network organization in relation to hemidesmosomal adhesion examining the consequences of interfering with focal adhesion, actin filament polymerization and microtubule formation on keratin-hemidesmosome interaction.

Keratin intermediate filaments consist of equimolar amounts of type I and type II keratin polypeptides [5,12,13]. In contrast to actin and tubulin, keratin polypeptides spontaneously self-assemble in the absence of nucleoside triphosphates and chaperones into apolar, highly flexible, and elastic 8–12 nm filaments, which form bundles of variable thickness [14]. The molecular mechanisms determining keratin network morphogenesis and 3D arrangement remain unknown. Basal epidermal cells contain keratins 5 and 14, which have been shown to bind to the hemidesmosomal plakin family cytolinkers plectin 1a [15] and bullous pemphigoid antigen 1 (BPAG-1; also referred to as BPAG-1e or BP230) [16]. These linker molecules, in turn, facilitate the attachment to the hemidesmosome-specific α6β4-integrin heterodimers [6,17], which bind to laminin 332 in the basement membrane [18,19]. Type II hemidesmosomes of simple epithelia consist only of α6β4-integrin heterodimers and plectin 1a-attached keratins, whereas type I hemidesmosomes, which occur in basal cells of pseudostratified and stratified epithelia, contain additional proteins, such as BPAG-1, the tetraspanin CD151, and the laminin 332-binding BPAG-2 (also referred to as BP180 or collagen type XVII) [6,20]. Recent studies suggest that type II hemidesmosomes mature into type I hemidesmosomes [21].

The function and distribution of hemidesmosomes in physiological and pathological in vivo situations is poorly characterized [22]. But it is known that hemidesmosomes are abundant in various epithelial tissues throughout the animal kingdom [23,24,25,26,27]. Grazing sections revealed that hemidesmosomes are regularly arranged as linear arrays (e.g., References [25,26,28]) reminiscent of the “beads-on-a-string” pattern described in recent in vitro studies [21,29], whereby keratin filaments (i.e., the “string”) interconnect hemidesmosomes (i.e., the “beads”) as is the case for desmosomal keratins [30]. The role of hemidesmosomes in wound-healing has also been cursorily investigated in vivo demonstrating that they are involved in the tongue-formation of keratinocytes invading the wound bed [31]. In the context of carcinogenesis, disassembly of hemidesmosomes and alternative association of the α6β4 integrin dimer with the actin cytoskeleton has been observed (cf. Reference [32]). Ultrastructural characterization of hemidesmosomes in invading tumor tissue, however, is scarce [33,34].

The molecular composition of actin stress fiber bundle-associated focal adhesions, which also form prominent attachment sites to the ECM, is fundamentally different from that of hemidesmosomes (review in References [35,36]). The anchorage of focal adhesions to the ECM is mediated through integrin-dimers, such as α2β1, α3β1, and α9β1, that are expressed in intact skin or α5β1, αVβ5 and αVβ6 that are upregulated during wound healing (cf. Reference [37]). Linker molecules between these integrins and the actin cytoskeleton include talin and vinculin [38,39]. Additional molecules with signaling functions, such as paxillin, focal adhesion kinase, and Src, are also recruited to focal adhesions [40,41,42].

Focal adhesions perform central functions in force transmission during cell migration [35]. The function of hemidesmosomes is less well defined. But an intricate interplay between focal adhesions and hemidesmosomes appears to take place during migration, which is of relevance during wound healing in human skin [29,31,32,37]. Even less is known about the coordination of the ECM-dependent organization of the different cytoskeletal systems although ECM-dependent coordinated regulation of cytoskeletal network dynamics in epithelial cells has been reported [43,44]. Most recently, a force-dissipating function has been assigned to hemidesmosomes and their associated keratin filaments by demonstrating that prevention of α6β4 adhesion increases focal adhesion-mediated and actin-dependent traction force generation in human keratinocytes [45]. A major impediment to more detailed analyses of the cross talk between focal adhesions and hemidesmosomes in the context of cytoskeletal network organization has been the lack of bona fide hemidesmosomes in many epithelial cell lines grown on glass. It has been shown, however, that immortalized and primary keratinocytes form cell adhesions preferably on laminin 332-coated surfaces that contain α6β4-integrins, together with plectin 1a, BPAG-1, and BPAG-2, serving as attachment sites for keratin intermediate filaments (e.g., References [21,29]). Keratin filaments and keratin filament bundles typically associate laterally with multiple hemidesmosomes [21,29]. These hemidesmosomal contacts are in close vicinity to focal adhesions indicative of structural, functional, and molecular links between both [6,29,45,46]. The precise nature of these links, however, and how they support the organization of the attached filament systems remains to be elucidated. 

Using the immortalized human keratinocyte cell line HaCaT, we show by high resolution microscopy of fixed immunocytochemical samples and vital cells producing fluorescent reporters that keratin filaments attach to hemidesmosomes, which are located in close proximity to focal adhesions. Treating these cells with inhibitors of actin and tubulin polymerization leads to loss of actin filaments, microtubules, and focal adhesions, while hemidesmosome-like entities with adhering keratin filaments remain. At the same time, the mesh size of the keratin cytoskeleton increases which is accompanied by bundling of keratin filaments. These findings demonstrate that established hemidesmosomes persist in the absence of focal adhesions and continue to serve as organizational hubs for the keratin system. Notably, keratin filament formation at hemidesmosomes is detected in this situation and also in migrating keratinocytes. The observed nucleation of growing keratin filaments that interconnect hemidesmosome assemblies is comparable to the recently described formation of keratins at desmosomal cell-cell adhesions [30]. Taken together, our findings confirm known and identify hitherto unknown features of the keratin-hemidesmosome system. We propose that these features confer special mechanophysical resilience and plasticity to epithelia against extreme stresses and strains that cannot be absorbed by the actin- and tubulin-based cytoskeletal components. Situations of extreme mechanical stress are particularly obvious in the epidermis. The epidermis is subjected to substantial pressure, especially the body weight-bearing foot sole epidermis, and is subjected to considerable viscous shear and tensile force as is the case for palmar epidermis of high bar gymnasts. These properties are reflected by epithelial superelasticity that has been recently linked to keratin intermediate filaments [47].

## 2. Results

### 2.1. Keratin Filaments Selectively Attach to and Interconnect Type I Hemidesmosomes That Are Juxtaposed to Focal Adhesions

**Figure 1:**
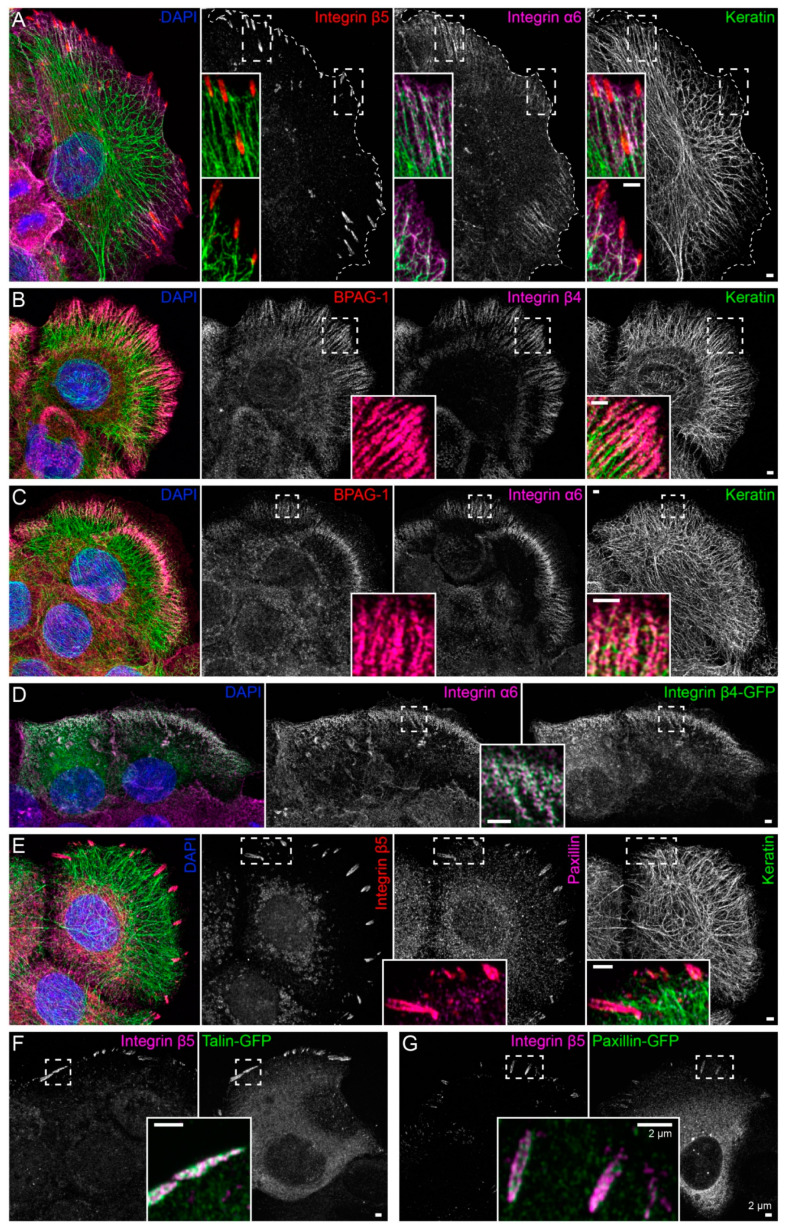
Keratin filaments are connected to hemidesmosomes, many of which are clustered next to focal adhesions. The fluorescence micrographs show antibody staining, nuclear 4′,6-diamidino-2-phenylindole (DAPI) staining (blue), and fluorescent reporters (at right in (D),(F),(G)) in methanol-fixed human keratinocytes (HaCaT). Enlargements of areas delineated by broken lines are shown as inserts. (**A**) The immunofluorescence images illustrate that the tips of keratin filament bundles localize next to but do not emanate from integrin β5-positive focal adhesions. Instead, peripheral keratin filaments co-localize with and connect integrin α6-positive hemidesmosomes (*n* = 8). (**B**,**C**) The pictures depict the co-distribution of bullous pemphigoid antigen 1 (BPAG-1) with integrin β4 and integrin α6 identifying the triple-positive structures as type I hemidesmosomes. Note, that they co-localize with peripheral keratin filament bundles, which are oriented along these extracellular matrix (ECM) adhesion sites (*n* = 7 and *n* = 11). (**D**) The images show two cells that were transfected with integrin β4-green fluorescent protein (integrin β4-GFP), which co-localizes with endogenous integrin α6 (*n* = 5). (**E**) The micrographs illustrate the co-distribution of integrin β5 with paxillin in focal adhesions and the lack of co-localization of focal adhesion markers with keratin filaments (*n* = 9). (**F**,**G**) The fluorescence recordings demonstrate that the fluorescent reporters talin-GFP and paxillin-GFP co-localize with integrin β5 in focal adhesions (*n* = 5 and *n* = 1). The image panels in (**A**–**D)** are maximum intensity projections of complete cells, and the image panels in (**E**–**G)** depict only the lower cell sections (also maximum intensity projections), where focal adhesions and hemidesmosomes are located.

Immortalized human HaCaT keratinocytes were used to investigate interactions between the keratin cytoskeleton and ECM adhesions. HaCaT cells form a monolayer under standard cell culture conditions. Cells were grown for 3 days to ≈90% confluency on glass that had been pre-coated with a laminin 332-rich matrix. This favored the formation of hemidesmosomes and ensured that cells were extremely flat and, therefore, ideally suited for high-resolution microscopy. Peripheral keratin filaments were often but not always detected next to clustered integrin β5, which is a component of focal adhesions (Figure 1A). In contrast, hemidesmosomal integrin β4- and BPAG-1-containing complexes co-localized with keratin filaments in a “beads on a string” pattern (Figure 1B). This pattern was also detected with antibodies directed against integrin α6, BPAG-1 and keratin (Figure 1C). The integrin α6/β4- and BPAG-1-positive structures can, thus, be classified as type I hemidesmosomes. Conversely, the integrin β5-positive structures were also positive for paxillin, identifying them as focal adhesions (Figure 1E).

To enable monitoring of the two types of ECM adhesions in living cells, fluorescent reporters were introduced into HaCaT keratinocytes by transfection of cDNA constructs. Hemidesmosomes were reliably labeled with integrin β4-green fluorescent protein (integrin β4-GFP) chimeras (Figure 1D), and either talin-GFP or paxillin-GFP reporters demarcated integrin β5-positive focal adhesions (Figure 1F,G).

### 2.2. Hemidesmosomal Keratin Filament Anchorage Persists in the Absence of Actin Filaments and Microtubules

**Figure A1:**
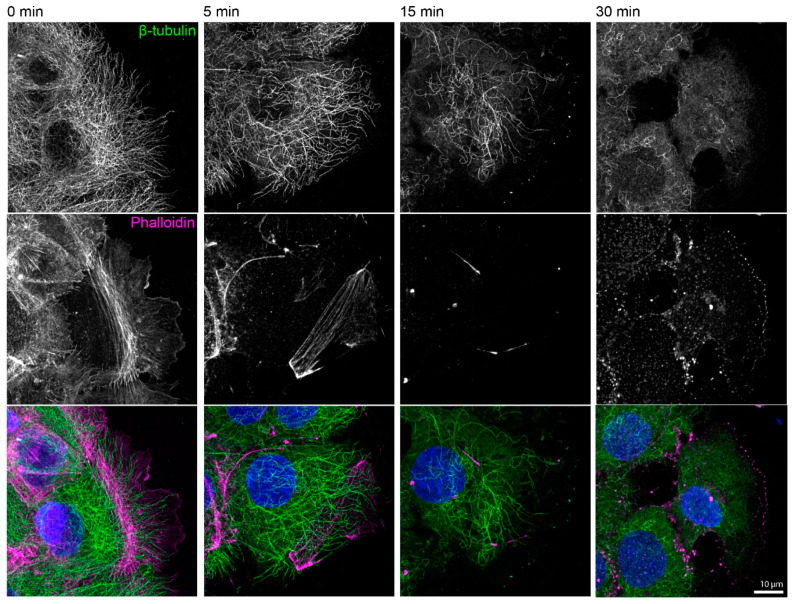
Actin filaments and microtubules are efficiently disassembled in the presence of 3 µM latrunculin B and 20 µM nocodazole. The fluorescence micrographs show the immunodetection of β-tubulin, together with phalloidin staining of filamentous actin and DAPI staining of nuclei (blue), in paraformaldehyde-fixed HaCaT keratinocytes before and 5, 15, and 30 min after inhibitor treatment. The cells were grown for 3 days to complete confluence, and the experiment was performed 1 h after a scratch was induced with a 20 µL pipette tip. All images are maximum intensity projections of entire cells (*n* = 4).

**Figure A2:**
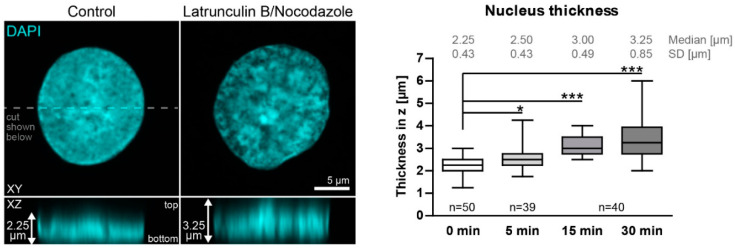
Nuclei enlarge in Z upon actin and microtubule inhibition. The fluorescence images depict a DAPI-stained nucleus of a methanol fixed HaCaT cell without any added inhibitor at left (Control) in comparison to that of a HaCaT cell that had been treated for 30 min with 3 µM latrunculin B and 20 µM nocodazole in XY (top) and XZ (bottom). To measure nucleus thickness, confocal planes with DAPI signal were identified, and the distance from the lowest to the highest plane was measured (2.25 µm versus 3.25 µm in the depicted examples). Note that it is not necessary to correct for skewness, since the bottom signal is mainly flat because of the glass surface below. The whisker box plot (whiskers from minimum to maximum; three independent experiments on different days; *n* values corresponding to cell numbers) on the right reveals that nuclear thickness increases significantly during inhibitor treatment. Kruskal–Wallis test is significant with a *p* < 0.0001 and Dunn’s multiple comparison post-test shows significant differences as indicated in the graph (*p < 0.05, ***p < 0.0001).

To investigate the contribution of actin filaments and microtubules to the keratin-hemidesmosome scaffold, HaCaT keratinocytes were treated with latrunculin B and nocodazole at different concentrations. Latrunculin B induced a dose-dependent inhibitory effect on mApple-actin polymerization as shown for 1 and 2 µM in time-lapse recordings (Video S1). For further experiments, a concentration of 3 µM was used to ensure reliable inhibition of actin polymerization. Twenty micromolar nocodazole treatment resulted in drastically reduced microtubule plus end-binding protein 3-green fluorescent protein (EB3-GFP) dynamics within seconds after inhibitor addition (Video S2; for efficient inhibitory effects of 33 µM nocodazole in HaCaT keratinocytes, also see References [48,49]). In further experiments, both inhibitors were added simultaneously to cells and samples were immunostained at different time points. Minor alterations in the architecture of the microtubule system became evident 5 min after drug addition, as determined by β-tubulin immunostaining. After 15 min, several microtubules were still detected and only very few scattered microtubules remained after 30 min (upper panels in Figure A1). In comparison, phalloidin staining revealed alterations in the architecture of the actin filament system 5 min after drug addition, near complete loss of actin filaments after 15 min, and the formation of differently sized granules after 30 min (middle panels in Figure A1). We, therefore, concluded that the chosen drug concentrations are highly efficient in disrupting both the actin and microtubule cytoskeleton within 15–30 min. We, furthermore, noted that a 30 min treatment with both drugs inhibited filopodia, lamellipodia, and ruffle formation and prevented cell migration. Cells instead retracted and rounded up, leading to increased cell height, which was reflected by expansion of the nucleus in the z-direction (Figure A2). We interpret this as a consequence of overall cell relaxation because of reduced intracellular tension.

**Figure 2:**
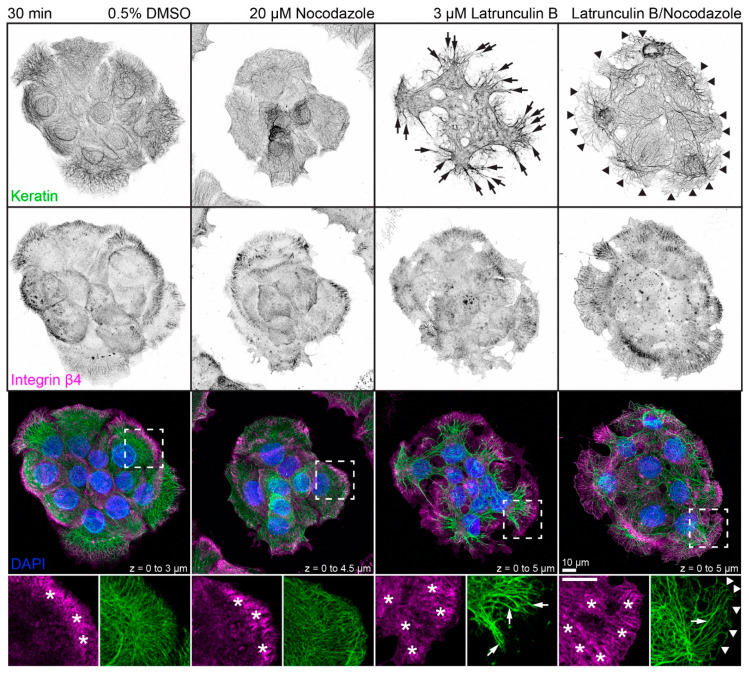
Disruption of actin filaments and microtubules alone or in combination leads to different changes in keratin filament network architecture in each situation. The triple fluorescence micrographs (enlargements of boxed areas in the bottom panel) show the immunodetection of keratins (top panel) and integrin β4 (middle panel), together with nuclear DAPI (merged images in lower panel), in methanol-fixed HaCaT keratinocytes. The actin cytoskeleton was disrupted by treating cells with 3 µM latrunculin B and the microtubule cytoskeleton was disrupted by treating cells with 20 µM nocodazole for 30 min. The dimethyl sulfoxide (DMSO) control on the left shows examples of typical keratin networks and hemidesmosomal distribution in untreated HaCaT cells. Note that the integrin β4-demarcated hemidesmosomal patterns (asterisks) are not affected by drug treatment, for the most part. On the other hand, major keratin filament network retraction is seen in latrunculin B treated cells (arrows), which is less pronounced in latrunculin B/nocodazole-treated cells (arrows) and not apparent in cells treated with nocodazole alone. Note also that prominent keratin bundles are present at the outermost margin of peripheral cells treated with both drugs (arrowheads) but not in untreated cells and cells treated with only one drug. All images are maximum intensity projections of entire HaCaT keratinocytes (*n* ≥ 10).

**Figure A3:**
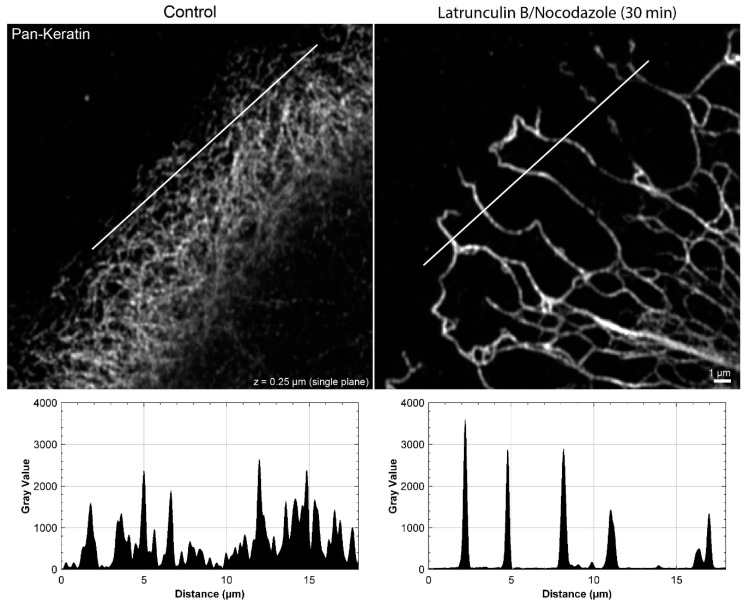
The peripheral keratin filament network completely re-organizes in the presence of 3 µM latrunculin B and 20 µM nocodazole. The immunofluorescence images depict keratin filaments, which are detected with pan-keratin antibodies, in single confocal planes of methanol-fixed HaCaT keratinocytes without any inhibitor (left) or after a 30 min latrunculin B/nocodazole treatment (right). The line scan shows that the fine keratin network in control cells cannot be fully resolved, whereas the bundled and strongly fluorescent keratin filaments in the network with large mesh size of the inhibitor-treated cell can be easily delineated.

The next set of experiments concentrated on the response of the keratin/hemidesmosome scaffold to 3 µM latrunculin B and 20 µM nocodazole. Figure 2 depicts the distribution of integrin β4-positive hemidesmosomes and keratins in small cell clusters in the presence of the solvent, latrunculin B, nocodazole, or latrunculin B/nocodazole. Typical hemidesmosomal distribution patterns were detectable and most prominent at the cell bottom of peripheral cells in each situation. On the other hand, treatment with latrunculin B induced retraction and straightening of keratin filaments which, however, still remained anchored in the cell periphery. Treatment with nocodazole affected keratin network organization only mildly inducing a slight increase in keratin filament straightness but no obvious keratin filament retraction. Notably, treatment with both drugs induced yet a different keratin network phenotype (also see Figure A3), while hemidesmosomal distribution was not visibly affected. In the latrunculin B/nocodazole-treated cells keratin networks retracted less than in cells treated with latrunculin B alone. In addition, keratin bundles appeared near the margin of peripheral cells that were not seen in the other conditions. Common to all drug-treated cells were perinuclear keratin network accumulations, which were associated with radial keratin filaments. The differences in straightness suggested that the radial filaments may be under tension, whereas the perinuclear filaments are relaxed. To study the dynamic nature of keratin network reorganization, time-lapse fluorescence microscopy was performed in HaCaT clone B10, which stably expresses keratin 5-yellow fluorescent protein (keratin 5-YFP) [50]. The recording presented in Video S3 confirmed the immunocytochemical observations. The keratin network retracted shortly after addition of both inhibitors but remained in part anchored in the cell periphery with straight and bundled filaments. Coarsening of mesh size and filament bundling occurred throughout the entire network with local granule formation in some, but not all, cells. The dynamic restructuring was best seen in the single bottom plane, revealing increasing filament bundling and ongoing filament formation in the cell periphery.

**Figure 3:**
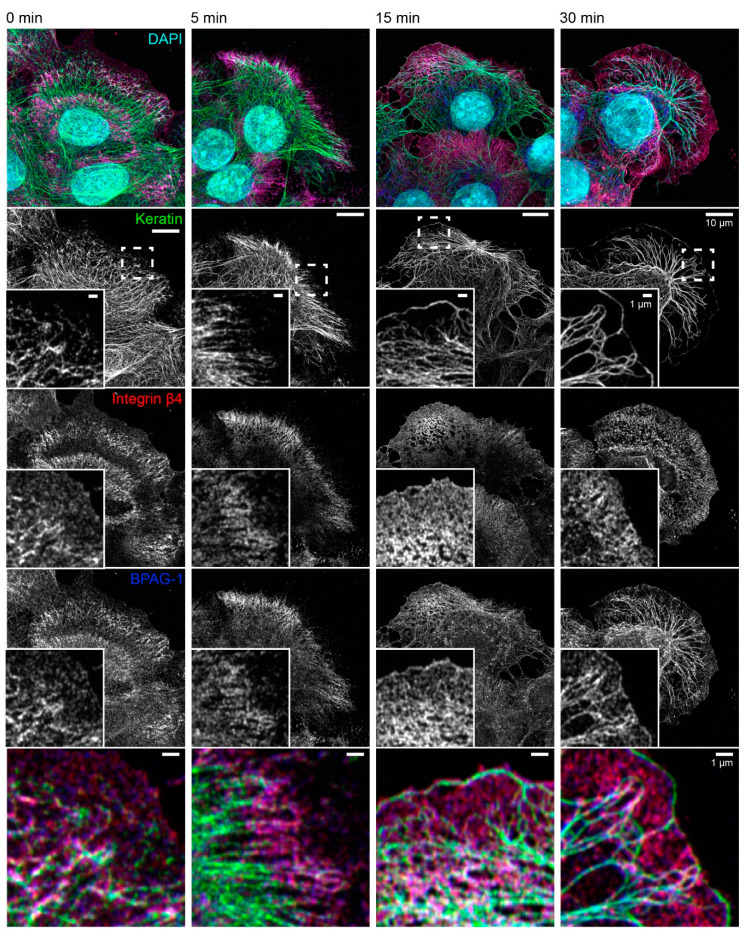
The keratin filament network retracts but remains attached to hemidesmosomes through straightened and bundled radial keratin filaments after actin filament and microtubule disruption. The quadruple fluorescence micrographs show the immunodetection of keratin, integrin β4 and BPAG-1, together with nuclear DAPI, in methanol-fixed HaCaT keratinocytes before and 5, 15, and 30 min after treatment with 3 µM latrunculin B and 20 µM nocodazole. The insets depict the areas marked by broken lines at higher magnification and as merged images in the bottom panel. Note that keratin filament bundles are attached to integrin β4-positive and BPAG-1-positive hemidesmosomes before treatment. The bulk of the keratin network retracts within 5 min of drug addition, while remaining attached to hemidesmosomes in the cell periphery. After 15 min, the keratin network is composed of thicker keratin filament bundles and has a larger mesh size. Additionally, new keratin filaments are detected in the juxtamembraneous domain co-localizing with integrin β4-positive and BPAG-1-positive clusters. A similar and even more pronounced change in keratin network organization is seen at time point 30 min with a few, hemidesmosome-anchored thick keratin bundles and long keratin filaments next to the plasma membrane. All images are maximum intensity projections of entire HaCaT keratinocytes (*n* ≥ 7).

**Figure 4:**
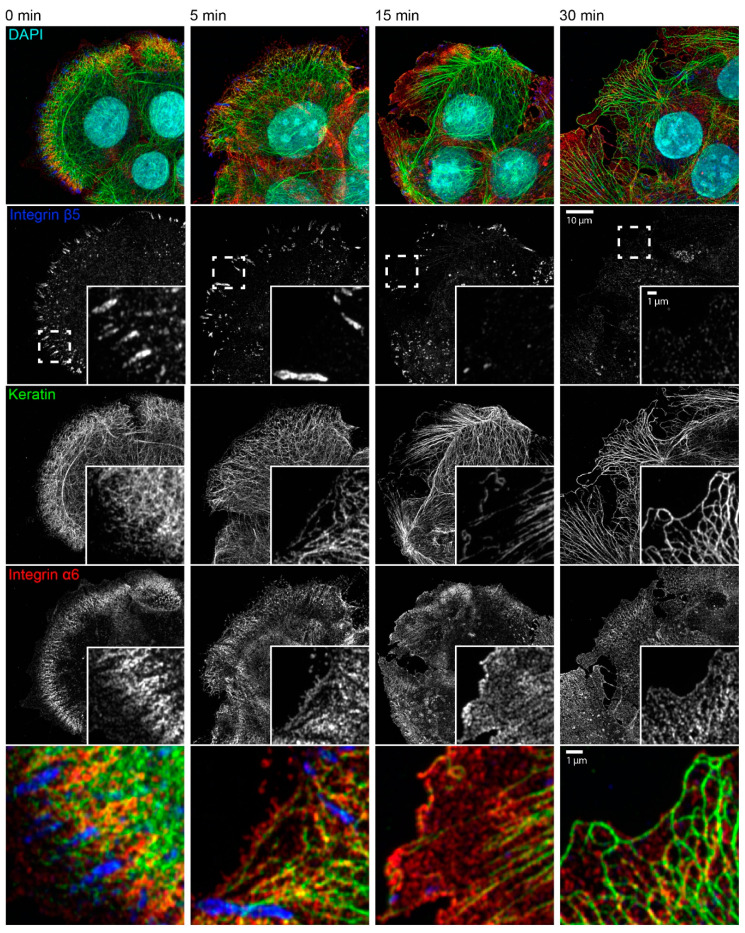
Keratin network reorganization after actin filament and microtubule disruption is not dependent on focal adhesions. The microscopic fluorescence images show the immunodetection of integrin β5, keratin, and integrin α6, together with nuclear DAPI staining, in methanol-fixed HaCaT keratinocytes that were treated with 3 µM latrunculin B and 20 µM nocodazole for different time periods. The inserts present the boxed regions (broken lines) at higher magnification. The bottom panel depicts merged images of the boxed regions. Before treatment, keratin filament bundles are attached to integrin α6-positive hemidesmosomal structures that are next to integrin β5-positive focal adhesions. Focal adhesions disassemble leading to granular integrin β5 clusters after 15 min and barely detectable clustered integrin β5 after 30 min of inhibitor treatment. At the same time, keratin filaments bundle, straighten, and retract toward the cell interior, while remaining attached to integrin α6-positive hemidesmosomes. Note that short keratin filaments elongate in the cell periphery, which become more prominent with time and co-localize with integrin α6 particles. All images are maximum intensity projections of entire cells (*n* ≥ 7).

The consequences of latrunculin B/nocodazole treatment for hemidesmosome-keratin distribution was assessed in more detail by immunocytochemistry in the next set of experiments (Figure 3). Five minutes after inhibitor addition, the keratin filament network retracted towards the cell center. It remained, however, attached to integrin β4-/BPAG-1-positive hemidesmosomes through radial, rather straight keratin filaments. After 15 and 30 min, increasingly thicker keratin filament bundles were detected, and the mesh size of the keratin filament network increased. At the same time, additional keratin filaments were detected at the outermost cell periphery next to weakly fluorescent integrin β4-/BPAG-1-positive dotted structures. In some cells, these filaments formed a continuous rim adjacent to the plasma membrane interconnecting the integrin β4/BPAG-1 clusters. Antibodies against integrin α6 and keratin confirmed the findings obtained for anti-integrin β4/BPAG-1/keratin immunostainings (Figure 4).

Taken together, we conclude that type I hemidesmosome-dependent keratin network organization persists after depletion of actin filaments and microtubules, resulting, however, in a novel keratin filament network phenotype.

### 2.3. Focal Adhesions Are Not Essential for the Maintenance of Hemidesmosomal Keratin Filament Anchorage

**Figure A4:**
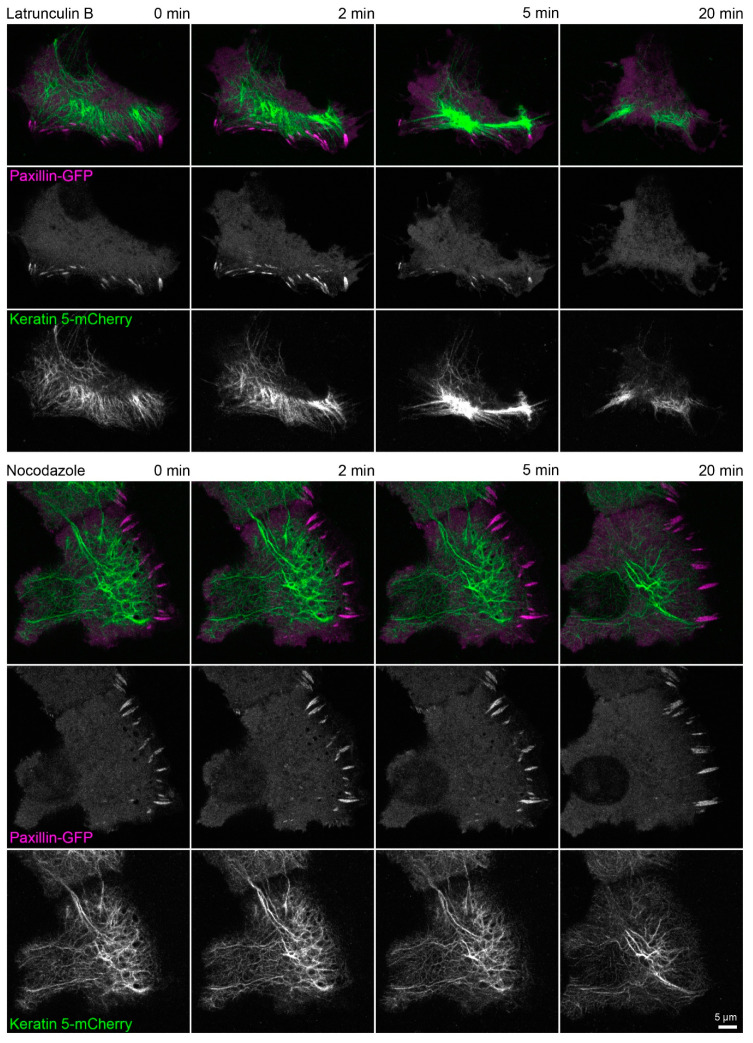
Actin polymerization is necessary for focal adhesion maintenance and assembly. The fluorescence images show the distribution of keratin 5-mCherry and paxillin-GFP in living HaCaT cells before and after treatment with 3 µM latrunculin B (upper part) or 20 µM nocodazole (lower part). Focal adhesions are disassembled within 20 min in the cell treated with latrunculin B and the keratin network strongly retracts but remains partially attached in cell periphery. On the other hand, focal adhesions in the cell treated with nocodazole persist and a delicate keratin network is still present after 20 min. All fluorescence images are maximum intensity projections of lower focal planes (*z* = 0 to 1.05 µm; 4 planes) (*n* = 4 and *n* = 3). The regions where focal adhesions are mainly located correspond to the cell colony edge. Both time-lapse series are shown in Video S3.

We next wanted to find out how the latrunculin B/nocodazole treatment affected focal adhesions in HaCaT cells. Immunocytochemistry showed that integrin β5-positive focal adhesions disassembled for the most part within 15 min and were virtually undetectable after 30 min (Figure 4). These findings were confirmed by time-lapse fluorescence microscopy of living cells, which also showed that latrunculin B affected focal adhesions but nocodazole alone did not (Figure A4 and corresponding Video S4).

**Figure 5:**
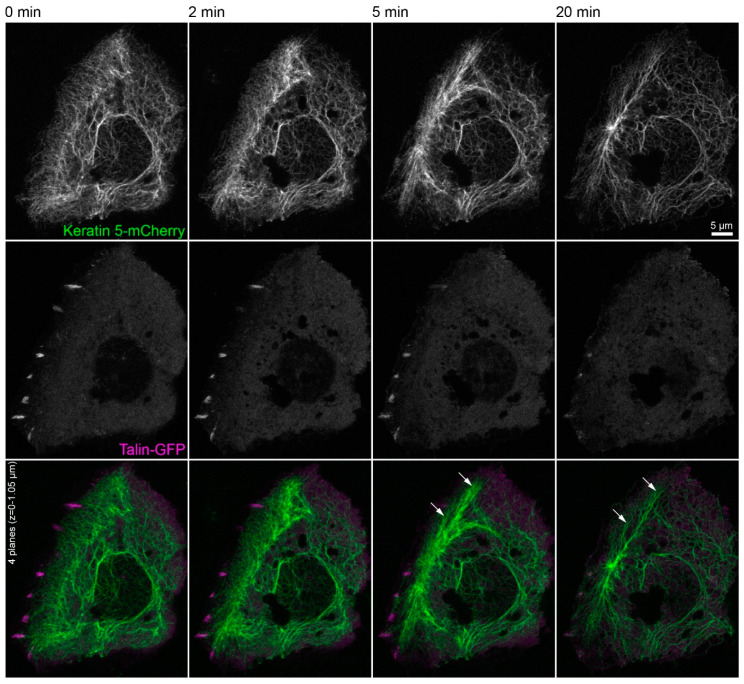
Keratin filament network reorganization is independent of focal adhesions in the absence of actin filaments and microtubules. The images depict keratin 5-mCherry (top) and talin-GFP fluorescence (middle; merge at bottom) in a vital HaCaT keratinocyte before and after treatment with 3 µM latrunculin B and 20 µM nocodazole. The keratin network retracts, bundles, and remains partially attached in the cell periphery (arrows) independent of the continued, though diminishing or even completely vanishing talin-GFP-fluorescence in some regions. All images are maximum intensity projections of the lower focal planes, as annotated in the figure (*n* = 3). Note that the cell is next to non-transfected, i.e., non-fluorescent cells. The complete image series is provided in Video S5.

The triple immunofluorescence micrographs in Figure 4 demonstrate that areas devoid of integrin β5-containing focal adhesions still maintained clustered integrin α6, which co-localized with keratin filaments. Time-lapse fluorescence microscopy of HaCaT cells co-expressing talin-GFP and keratin 5-mCherry confirmed these observations (Figure 5 and corresponding Video S5). They also supported the above-mentioned alterations in keratin filament organization and provided additional details. This included the retraction of the keratin network toward the nucleus, the bundling of keratin filaments coincident with network coarsening, and the appearance of filament bundles in the cell periphery, presumably at hemidesmosomal adhesions. In addition, the overall motility of the keratin cytoskeleton, including that of small keratin particles, ceased almost completely.

### 2.4. Keratin Filament Nucleation Occurs at Newly-Formed Hemidesmosomes and Does Not Require Actin Filaments and Microtubules

**Figure 6:**
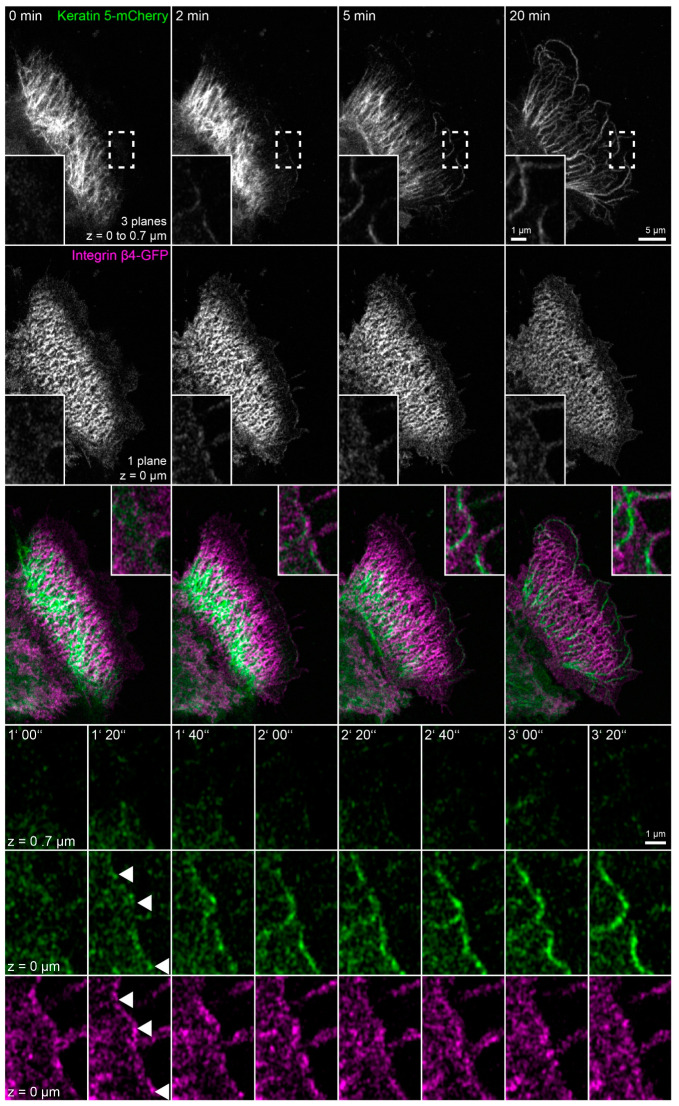
Growing keratin filaments appear at nascent hemidesmosomes after actin and microtubule disruption (*n* = 6). The fluorescence images show the distribution of keratin 5-mCherry and integrin β4-GFP in a living HaCaT cell before and after treatment with 3 µM latrunculin B and 20 µM nocodazole. The images of keratin 5-mCherry fluorescence present maximum intensity projections of the lower focal planes, and the images of the integrin β4-GFP fluorescence show only the bottom focal plane. The boxed regions are depicted at higher magnification in the insets and at higher temporal and spatial resolution in the bottom panels to illustrate ongoing keratin filament nucleation and growth at integrin β4-positive clusters in the cell periphery. As a result, new keratin filament bundles appear at the cell edge that co-localize and connect newly-formed integrin β4-GFP complexes. Note that the weak integrin β4-GFP signal decreases significantly because of bleaching. The images also show that the drug treatment induces keratin network retraction and bundling, while keratin filament bundles remain attached to hemidesmosomal structures. The fluorescent cell is adjacent to non-transfected cells. The complete time-lapse recording is provided in Video S6.

To directly examine the dynamic behavior of hemidesmosomes and keratins in latrunculin B/nocodazole-treated HaCaT cells and, specifically, to determine the origin of the peripheral keratin filaments, keratin 5-mCherry and integrin β4-GFP were imaged by time-lapse fluorescence microscopy (Figure 6 and corresponding Video S6). Two minutes after addition of the inhibitors, the keratin network started to retract, and keratin filaments bundled, extending from the nucleus towards the cell periphery. The peripheral parts of these filaments co-localized with integrin β4 and remained attached throughout the observation period. It, furthermore, appeared as if the retracting keratin filaments transmitted force on the hemidesmosome-anchored keratin filaments, as evidenced by their straightening and radial orientation, with respect to the nucleus-containing cell center. Noticeably, new integrin β4 clusters appeared at the plasma membrane. Unfortunately, these initially rather weak fluorescence signals bleached considerably during the recordings. But it was evident that they served as nucleation sites for novel keratin filaments, which subsequently elongated locally (enlargements at the bottom of Figure 6). These keratin filaments successively connected to establish the peripheral submembraneous scaffold that had been detected by immunocytochemistry (Figure 3 and Figure 4). A likely explanation for this finding is that keratin filament nucleation proceeds in the absence of actin filaments and microtubules, resulting in keratin filament self-assembly next to nucleation sites without further intracellular distribution because of inactivation of actin filament- and microtubule-dependent transport systems.

### 2.5. Keratin Filaments Nucleate at Nascent Hemidesmosomes in Migrating Cells

**Figure 7:**
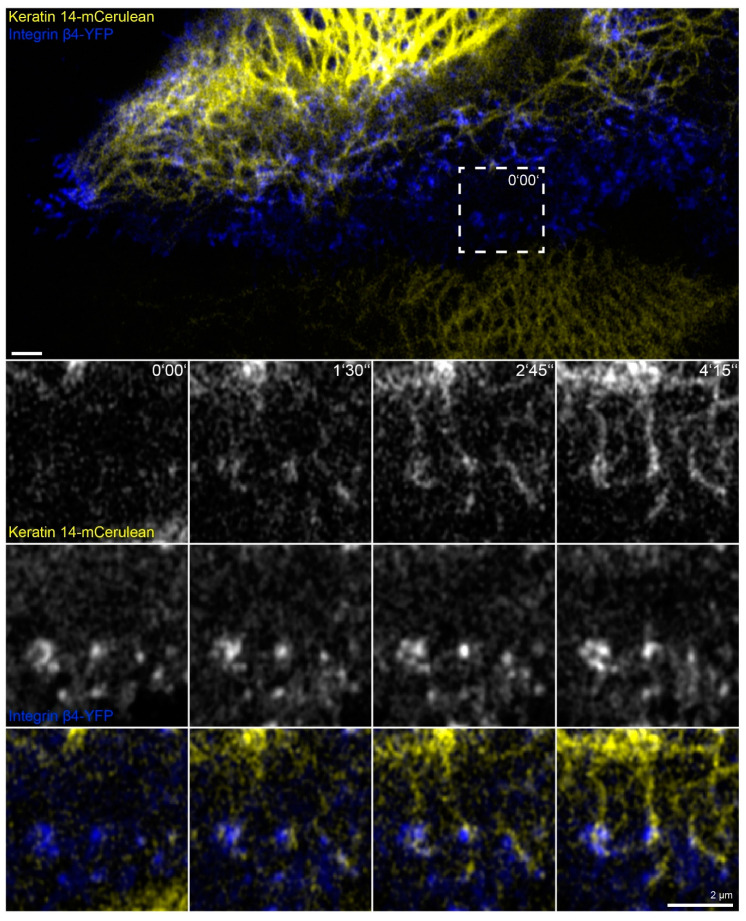
Keratin filaments are assembled at and interconnect hemidesmosomes in migrating cells. The fluorescence images depict the distribution of keratin 14-mCerulean and integrin β4-yellow fluorescent protein (integrin β4-YFP) in a migrating HaCaT cell that is next to another cell, which is only positive for keratin 14-mCerulean. Note that both cells are surrounded by non-transfected cells. The magnified time series recorded in the square delineated by a broken line shows different stages of keratin filament assembly at nascent hemidesmosomal integrin β4-YFP clusters. The growing filaments interconnect hemidesmosomes and gain contact with the peripheral keratin cytoskeleton. All images were recorded at the bottom focal plane (n = 20, all cells were migrating). The entire time-lapse series is shown in Video S7.

In this set of experiments, we wanted to find out whether the phenomena detected in the absence of actin filaments, microtubules and focal adhesions are also of relevance for cells containing these cytoskeletal components. To increase hemidesmosome formation, cells were grown to complete confluence, and migration was induced by a scratch with a 20 µL pipette tip. The wounded cell-layers typically started to close the wound within minutes. During migration of the cells, new focal adhesions and hemidesmosomes were formed at the leading edges and were disassembled at the trailing edges as previously described for migrating primary keratinocytes [29]. At the same time, new keratin filaments were formed preferentially at the leading edge that subsequently gained contact to the main cytoskeletal keratin network (Figure 7 and corresponding Video S7; also see References [44,51]). Shortly before the appearance of keratin particles, integrin β4-YFP clusters could be detected at the same positions. These nucleating keratin particles remained attached to the hemidesmosomal clusters. The nascent keratin particles subsequently elongated in one direction and interconnected hemidesmosomal protein clusters. The newly formed scaffold finally connected to pre-existing filaments of the more central keratin filament network.

## 3. Discussion

Keratin network organization in the combined absence of actin filaments and microtubules has rarely been investigated. Wöll et al. [10] showed that simultaneous inhibition of actin and microtubule polymerization results in a complete collapse of the keratin filament network in non-epithelial, adrenal cortex-derived SW13 cells producing fluorescence-tagged keratins 8 and 18. In contrast to those findings, we show that the keratin 5 and 14 positive filament network retracts towards the nucleus but remains partially extended under comparable conditions in epithelial HaCaT keratinocytes. The obvious reason for this difference is the presence of hemidesmosome-like structures in HaCaT cells and their absence in SW13 cells. This finding emphasizes the importance of hemidesmosomal anchorage for keratin network organization.

**Figure 8:**
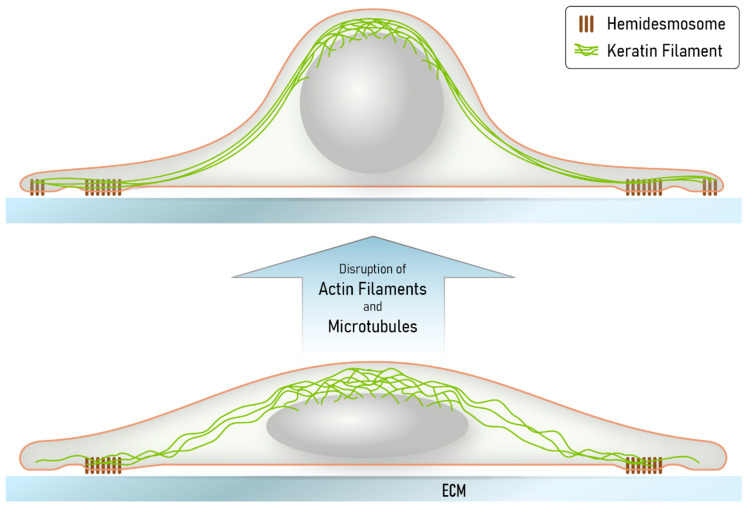
The scheme summarizes our major findings and illustrates how the keratin network may provide mechanical resilience to extreme cellular deformation, leading to network reorganization through filament bundling and filament nucleation at nascent hemidesmosomes. Disruption of both the actin filament and microtubule systems induces cell shape changes because of loss of cortical actomyosin contractility and altered intracellular stiffness. Together, these changes are predicted to lead to a rounding of the nucleus and the surrounding cell body, which is counterbalanced by the keratin intermediate filament cytoskeleton. Since the hemidesmosome-anchored keratin filament network persists, radial filaments become stretched, losing their waviness and forming thick bundles. Localized hemidesmosome formation and keratin filament nucleation allows adjustment of the remaining cytoskeleton to cell shape. We propose that deformations, which may, for example, occur in migrating cells after wounding or in epidermal cell layers subjected to tensile stress and viscous shear stress, elicit comparable responses in the superelastic keratin intermediate filament network.

We further suggest that the obviously non-physiological situation created in our cultured cells may be of relevance for certain in vivo situations, including, for example, conditions of extreme stretch or at the tips of collectively migrating cells during wound closure or invading tumor cells, when the hemidesmosome/desmosome-keratin system kicks in because of insufficiency of the other less extensible and flexible cytoskeletal networks [47,52]. The tensegrity model proposes that the acto-myosin and microtubule-motor protein systems are important determinants of cell shape [53,54]. Their depletion releases these constraints and cells are expected to round up. This is exactly what we observed in the latrunculin B/nocodazole-exposed HaCaT cells. The remaining hemidesmosome-anchored keratin filaments that are connected to the perinuclear cage are thereby stretched. At the same time, the finely grated keratin network lattice slides and compacts into thick filament bundles. This idea is depicted in the scheme in Figure 8.

Our observations also show, for the first time, that keratin filaments nucleate at hemidesmosomes. The proximity of hemidesmosomes to focal adhesions explains our previous observations, which identified focal adhesions as “hotspots” of keratin filament formation [55]. In contrast to vimentin, however, keratins do not bind to focal adhesions [56,57]. The high-resolution images in the present study demonstrate that keratin filaments nucleate and grow from hemidesmosomes where they remain anchored. The nucleating and growing hemidesmosome-attached keratin particles are reminiscent of the previously described motile keratin particles, which have been referred to as keratin filament precursors [58,59] or keratin squiggles [11].

The current findings of keratin nucleation and growth at hemidesmosomes are strikingly similar to those reported recently for desmosomes [30]. In both instances, nascent spheroidal keratin particles are first detected next to clustered transmembrane adhesion receptors, i.e., β4 integrins and desmosomal cadherins, respectively. These particles elongate into small rodlets that grow unidirectionally and interconnect adjacent adhesion clusters forming submembraneous keratin lattices. Filaments bundle and connect to the remaining network. The fact that keratin nucleation occurs in these molecularly distinct desmosomal and hemidesmosomal domains suggest that common mechanisms apply. Thus, recruitment of plakin-domain proteins, i.e., plectin 1a and BPAG-1 at hemidesmosomes and desmoplakins at desmosomes, leads to an increase of keratin polypeptides, which have been shown to bind to plakin domains [15,16,60]. The local increase in keratins may suffice to exceed a threshold level needed for spontaneous keratin assembly. The same mechanism may apply to the recently reported recruitment of keratins to apical microridges [61].

Taken together, our findings assign important mechanical functions on the keratin-hemidesmosome scaffold, which is predominant in basal cells of homeostatic epidermis reacting dynamically to wounding by localized reformation and providing protection against extreme mechanical stress, resulting in deformation that cannot be absorbed by actin filaments and microtubules.

## 4. Materials and Methods

### 4.1. Cell Culture

Immortalized human HaCaT keratinocytes were kindly provided by Dr. Petra Boukamp [62] and were grown at 37 °C in a 5% CO_2_ humidified atmosphere and Dulbecco’s Modified Eagle’s Medium (DMEM) containing l-alanyl-glutamine (Sigma-Aldrich, St. Louis, MO, USA) and 10% (*v/v*) fetal bovine serum (FBS) SeraPlus (PAN Biotech, Aidenbach, Germany). For passaging, cells were washed and incubated for 15 min in phosphate-buffered saline (PBS) without Ca^2+^/Mg^2+^ (Sigma-Aldrich). They were thereafter trypsinized for ≈5 min in a solution of PBS without Ca^2+^/Mg^2+^ (Biochrom, Schaffhausen, Switzerland) containing 0.2% (*w/v*) trypsin (Biochrom) supplemented with 0.02% (*w/v*) EDTA (Sigma-Aldrich). Cells were passaged once per week one day after reaching confluence and were seeded at a concentration of 40,000–60,000 cells/cm^2^ in 6 ml cell culture medium in 25 cm^2^ cell culture flasks (Greiner Bio-One, Frickenhausen, Germany). For experiments, cells were grown in 35-mm diameter dishes at a concentration of ≈100,000 cells/cm^2^ with 2 ml cell culture medium. For immunocytochemistry, glass cover slips were precoated with laminin 332-rich matrix from 804G cells, as described in References [50,63]. For live cell microscopy, complete culture dishes were precoated with laminin 332-rich matrix.

Transfections were performed on day 2 after seeding by addition of 100 µL Xfect reaction buffer, together with 5 µg plasmid DNA and 1.5 µL Xfect (Takara Bio, Kusatsu, Shiga, Japan), according to the manufacturer’s protocol, but without medium removal. For simultaneous transfection with two plasmids, 2.5 µg DNA was used for each.

Latrunculin B (AdipoGen, Liestal, Switzerland) was dissolved in pure dimethyl sulfoxide (DMSO; Sigma-Aldrich) at a concentration of 1 mM and nocodazole (Sigma-Aldrich) at a concentration of 10 mM. Both stock solutions were stored at −20 °C for a maximum of 3 months, and thawed aliquots were used within one day. The final DMSO concentration after drug addition to cells was 0.5% (*v/v*).

Wound closure assays were performed by inducing a vertical scratch through confluent cell monolayers with a 20 µL pipette tip (Starlab International, Hamburg, Germany) and replacing the cell culture medium directly afterwards twice.

### 4.2. Immunocytochemistry

For immunocytochemistry, HaCaT cells were grown on 18-mm diameter high-precision glass cover slips with a thickness of 170 µm (Paul Marienfeld, Lauda-Königshofen, Germany) in six-well dishes (CytoOne®, Starlab International) for 3 days. Fixation was performed by incubation in fresh 99.9% (*v/v*) methanol (Alfa Aesar, Heysham, United Kingdom) for 3 min at −20 °C followed by washing in PBS (Biochrom) at room temperature for 5 min and optional storage over night at 4 °C. Alternatively, cells for actin and β-tubulin staining in Figure A1 were fixed in pre-warmed (37 °C) 4% (*v/v*) paraformaldehyde (Merck, Darmstadt, Germany) in PBS (pH 7.2–7.4; adjusted with NaOH at up to 60 °C) for 15 min at room temperature, and cell membrane permeabilization was accomplished by incubation for 3 min in 0.2% (*v/v*) Triton-X100 (Sigma-Aldrich) in PBS. Blocking of cells was performed with 5% (*w/v*) bovine serum albumin (BSA; SERVA, Heidelberg, Germany) in PBS for 1 h (except for cells depicted in Figure A1). Primary and secondary antibodies were diluted in 1% (*w/v*) BSA in PBS. The samples were incubated with primary antibodies for 1 h, washed with PBS for 5–15 min, and incubated with secondary antibodies and 0.2 µg/ml 4′,6-diamidino-2-phenylindole (DAPI; Hoffmann La Roche, Basel, Switzerland) for 40 min. Finally, cells were washed with PBS for 20 min and deionized H_2_O for 30 s before mounting with Mowiol (Carl Roth, Karlsruhe, Germany) on glass slides (R. Langenbrinck, Emmendingen, Germany). The prepared samples were dried over night at 4 °C and stored at the same temperature until recording, up to 2 weeks.

Guinea pig pan cytokeratin antibody cocktail (GP14) was from Progen Biotechnik (Heidelberg, Germany). Monoclonal rat anti-integrin α6 antibody (clone GOH3) was from R&D Systems (Minneapolis, MN, USA) and anti-integrin β4 (clone 439-9B) from BD Pharmingen (San Diego, CA, USA). Monoclonal mouse anti paxillin (clone 349) was from BD Transduction Laboratories (San Jose, CA, USA), anti-BPAG-1 (clone 279) from Cosmo Bio (Carlsbad, CA, USA), and anti-α-tubulin (clone DM1A) from Thermo Fisher Scientific (Waltham, MA, USA). Monoclonal rabbit anti integrin β5 (clone D24A5) was from Cell Signaling Technology (Danvers, MA, USA). Alexa Fluor 546 Phalloidin was from Thermo Fisher Scientific. Secondary antibodies coupled to the Alexa family of fluorophores were from Thermo Fisher Scientific, and secondary antibodies coupled to cy3 or DyLight550 were from Dianova (Hamburg, Germany). For combinations and concentrations of antibodies used, see Table A1.

### 4.3. Microscopy

Microscopical recordings were performed with a laser scanning confocal microscope (LSM 710) using Zen black 2.1 SP3 software (Carl Zeiss, Jena, Germany). The microscope was equipped with an Airyscan detector, oil immersion objective (63×/1.40-N.A., DIC M27), and a focus-shift correction system (DefiniteFocus; all from Carl Zeiss). For live-cell imaging, the microscope was pre-warmed to 37 °C in an incubation chamber. Living HaCaT cells were imaged in glass-bottom dishes (12 mm glass-diameter, thickness 1.5#, MatTek, Ashland, MA, USA) in 25 mM 4-(2-hydroxyethyl)-1-piperazineethanesulfonic acid-buffered DMEM without phenol red (Life Technologies, Carlsbad, CA, USA) supplemented with 2% (*v/v*) FBS. Fluorescent reporter protein dynamics were recorded at 16 bit-depth with the Airyscan detector in “resolution vs. sensitivity” mode, and the signal was processed with automatic 2D settings. The only exception was the recording in Video S2, where the detector was used in conventional mode for best incident photon to current efficiency. Immunostainings and reporter fluorescence in fixed cells were recorded in “super resolution” mode, and the signal was processed with 3D automatic settings.

An argon-ion laser (module LGK 7872 ML8) was used at 458 nm for detection of mCerulean, at 488 nm for GFP/Alexa488, and at 514 nm for YFP. For detection of mApple/mCherry/Alexa546/Alexa555/ DyLight550/cy3, a 543 nm HeNe-laser (module LGK 7786 P) was used. For detection of Alexa 647, a HeNe-laser (module LGK 7628-1F) was used, and, for detection of DAPI, a 405 nm diode laser (laser cassette 405 cw) was used. Live cell recordings of mCerulean, together with YFP, were performed at 465–505 nm for mCerulean signal; otherwise, filters were not needed to prevent noticeable signal bleed through resulting in faster acquisition speed. In immunostainings, DAPI was recorded at 420–480 nm, Alexa 488 at 460–480 nm and 495–550 nm, Alexa 546/555 and cy3 and DyLight550 at 570–620 nm and above 645 nm, Alexa 647 above 605 nm. In general, the detector gain was set at 850–900 for living cells and 750–850 for fixed samples. The samples were scanned at maximum speed at automatically calculated optimal resolution (except for cells depicted in Figure 2, where a less precise resolution of 2048 × 2048 pixels for 133 × 133 µm was used). The z-resolution was set to 0.35 µm for living cells and to 0.25 µm for fixed cells. The pinhole was set for all channels to a single value that was optimal (auto setting) for the green emission range.

### 4.4. Plasmids

Human keratin 14-mCerulean was described before in Reference [30]. Murine talin-GFP ((GFP)-talin) was a kind gift from Wolfgang Ziegler (then at University of Leipzig, Germany [64]). EB3-GFP (end-binding protein 3-GFP) was a kind gift from Rainer Duden (Universität zu Lübeck, Germany [65]). mApple-Actin was a kind gift from James Nelson (Stanford University, Stanford, CA, USA).

Human integrin β4-GFP (GFP-hβ4) was a kind gift from Jonathan C.R. Jones (then at Northwestern University, Evanston, IL, USA; [66]). We used this plasmid to generate a YFP version. To this end, an in frame YFP-encoding sequence from pEYFP-N1 (Clontech Laboratories, Mountain View, CA, USA) was cloned with primers 5′-ATTCAGGATCCATCGCCACCATGGTGAGCAAGGG-3′ and 5′-CAAATGTGGTATGGCTGATTATG-3′ and subcloned into pEYFP-N1 using BamHI/XbaI restriction sites, thereby substituting the original YFP-encoding cassette. Next, the YFP-encoding fragment was excised with NheI/KpnI and was used to replace the GFP-encoding part of GFP-hβ4, resulting in plasmid integrin β4-YFP.

Human keratin 5 (HK5) cDNA was kindly provided by Harald Hermann (German Cancer Research Center, Heidelberg, Germany). The keratin 5 sequence was copied with primers 5′-AAAAAGCTTATGTCTCGCCAGTCAAGTGTG-3′ and 5′-AAAGGATCCGGGCTCTTGAAGCTCTTCCGGGA-3′ and integrated into pECFP-N1 (Clontech Laboratories) using HindIII- and BamHI-restriction sites producing plasmid C-HK5-ECFP. In a next step, the ECFP-encoding part was replaced by a cDNA fragment encoding mCherry from pRSET-B mCherry (a kind gift from Roger Tsien, University of California, San Diego, CA, USA). To this end, the mCherry fragment was subcloned into pEYFP-N1 using BamHI/NotI. The fragment was then excised with XhoI/BamHI and integrated into C-HK5-ECFP after ECFP removal generating plasmid keratin 5-mCherry.

Paxillin-dsRed2 was a kind gift from Alan Rick Horwitz (University of Virginia School of Medicine, Charlottesville, VA, USA [67]) and was used to generate plasmid paxillin-GFP. In short, the paxillin cDNA was first subcloned into NdeI/XbaI of pLVX-Ires-puro (Clontech Laboratories) and then amplified from the plasmid DNA with primers 5′-TATGTCGACATGGACGACCTCGATG-3′ and 5′-ATAGGATCCGAGTTTGAGAAAGCAGTTCTG-3′. The amplified product was then further subcloned into the SalI/BamHI sites of pcBh_eGFP_T2A_Puro (a kind gift from Sebastian Kant) that is based on pSpCas9(BB)-2A-Puro (PX459) V2.0 (a kind gift from Feng Zhang; Addgene plasmid #62988). The plasmid lacks the U6 promoter and sgRNA sequences and contains the coding sequence for GFP in the multicloning site.

### 4.5. Image Processing, Data Presentation, and Statistics

Microscopic images were processed and analyzed with the open source Fiji distribution of the ImageJ software package [68,69], except maximum intensity projections and gamma adjustments in Video S3 that were performed with Zen 3.0 SR software (Carl Zeiss). Figures were prepared with Adobe Photoshop and Illustrator CS 6 (Adobe, San Jose, CA, USA). Movies were encoded in h.264 video format using Handbrake 1.3.3 open source software (https://handbrake.fr/) (accessed on 20 February 2021).

The presented immunostainings were selected from multiple high-resolution images (denoted as n in the figure legends) recorded from single glass cover slips. Note that the experiments shown in Figure 1, Figure 2, Figure 3, Figure 4, Figure 5 and Figure 6 and Videos S3–S6 were all done under identical standard conditions, differing only by the presence and time of inhibitor treatment and, thereby, serving as controls for each other. Furthermore, the experiments in Figure 2 and Figure 6 were performed twice, while experiments in Figure 7 were performed four times on different days.

Statistical analyses were performed with the help of GraphPad Prism 5.01 (GraphPad Software, San Diego, CA, USA).

## Data Availability

Not applicable.

## Supplementary Materials

Supplementary materials can be found at https://www.mdpi.com/1422-0067/22/4/2130/s1.

## Abbreviations

BSA	bovine serum albumin
ECFP	enhanced cyan fluorescent protein
DAPI	4′,6-diamidino-2-phenylindole
DMSO	dimethyl sulfoxide
EB3	end-binding protein 3
ECM	extracellular matrix
GFP	green fluorescent protein
FBS	fetal bovine serum
PBS	phosphate-buffered saline
YFP	yellow fluorescent protein

## Appendix A

**Table A1:** List of primary and secondary antibody combinations and concentrations.

Figure	Primary Antibody	Dilution	Secondary Antibody with Catalog Number	Dilution
Figure 1A,B,E, Figure 2, Figure 3, Figure 4 and Figure A3	Guinea pig pan anti-cytokeratin	1:200	Goat anti-guinea pig, Alexa fluor 488 (A-11073)	1:500
Figure 1C	Guinea pig anti-cytokeratin pan	1:200	Goat anti-guinea pig, Alexa fluor 647 (A-21450)	1:250
Figure 1, Figure 2 and Figure 3	Rat anti-integrin β4	1:200	Goat anti-rat, Alexa fluor 555 (A-21434)	1:500
Figure 1A,E and Figure 4	Rabbit anti-integrin β5	1:200	Goat anti-rabbit, Alexa fluor 647 (A-21245)	1:250
Figure 1F	Rabbit anti-integrin β5	1:200	Donkey anti-rabbit, cy3 (711-166-152)	1:500
Figure 1G	Rabbit anti-integrin β5	1:200	Donkey anti-rabbit, DyLight 550 (DkxRb-003-F550NHSX)	1:500
Figure 1B and Figure 3	Mouse anti-BPAG-1	1:150	Goat anti-mouse, Alexa fluor 647 (A-21236)	1:250
Figure 1C	Mouse anti-BPAG-1	1:150	Goat anti-mouse, Alexa fluor 488 (A-11029)	1:500
Figure 1 and Figure 4	Rat anti-integrin α6	1:200	Goat anti-rat, Alexa fluor 555 (A-21434)	1:500
Figure 1E	Mouse anti-paxillin	1:100	Goat anti-mouse, Alexa fluor 555 (A-21424)	1:500
Figure A1	Mouse anti-β-tubulin	1:300	Goat anti-mouse, Alexa fluor 488 (A-11029)	1:500
