# Supplementary material for: Hemidesmosome-Related Keratin Filament Bundling and Nucleation
Source: Int J Mol Sci. 2021 Feb 21;22(4):2130. doi: 10.3390/ijms22042130 (PMC7924876; doi:10.3390/ijms22042130)
Supplement: Supplementary file 1 [file ijms-22-02130-s001.zip › ijms-1070435- supp video xml.pdf]

Supplementary video explanations

Video S1. Treatment of HaCaT keratinocytes with latrunculin B disrupts actin dynamics within a few minutes in a dose-dependent manner. The video presents time-lapse fluorescence microscopy of HaCaT cells producing mApple-Actin before and after addition of either 2 µM or 1 µM latrunculin B. The cells treated with the higher concentration show a stronger decrease in actin-dynamics and a pronounced disassembly of actin structures. Note that there are adjacent non- or weakly-transfected cells. The recordings show only bottom focal planes. The original resolution was downscaled in the video with a bicubic filter to preserve detail.

Video S2. Treatment of HaCaT keratinocytes with 20 µM nocodazole stops microtubule dynamics instantaneously. The image series shows fluorescence recordings of EB3-GFP in HaCaT cells before and after addition of 20 µM nocodazole. The drug was washed out after 10 min incubation, resulting in slow re-assembly of EB3-GFP positive microtubules in the cell periphery. The recording shows maximum intensity projections of lower focal planes (z = 0 to 1.05 µm; 4 planes), and the signal was processed as annotated in the video.

Video S3. Simultaneous disruption of actin filaments (3 µM latrunculin) and microtubules (20 µM nocodazole) induces partial keratin network retraction, keratin filament bundling, and formation of new keratin filament bundles in the cell periphery near the cell bottom (compare recordings at z = 0 nm with those at z = 700 nm). The fluorescence images were recorded in HaCaT B10 cells producing fluorescent keratin 5-YFP.

Video S4. Treatment of HaCaT keratinocytes with latrunculin B disassembles focal adhesions, whereas nocodazole does not affect focal adhesions. The video shows time-lapse fluorescence recordings of paxillin-GFP and keratin 5-mCherry in HaCaT cells before and after addition of 3 µM latrunculin B (top panels) or 20 µM nocodazole (lower panels). The recordings show maximum intensity projections of lower focal planes (z = 0 to 1.05 µm; 4 planes). The original resolution was downscaled in the video with a bicubic filter to preserve detail. In addition see corresponding Figure A4.

Video S5. Keratin network reorganization is independent of focal adhesions in the absence of actin filaments and microtubules. The image series shows the fluorescence of keratin 5-mCherry and talin-GFP in a HaCaT cell of a monolayer that was treated with 3 µM latrunculin B and 20 µM nocodazole. The recording shows maximum intensity projections of lower focal planes (z = 0 to 1.05 µm; 4 planes) at original resolution. See corresponding Figure 5 for more details.

Video S6. Growing keratin filaments appear at nascent hemidesmosomes after actin and microtubule disruption. The image series shows the fluorescence of keratin 5-mCherry and integrin β4-GFP in a HaCaT cell of a monolayer that was treated with 3 µM latrunculin B and 20 µM nocodazole. See annotations in the video and corresponding Figure 6 for more details.

Video S7. Keratin filaments are assembled at and interconnect hemidesmosomes. The image series shows the fluorescence of keratin 14-mCerulean and integrin β4-YFP in a migrating HaCaT cell within a monolayer a few minutes after a scratch was induced. The recording shows the bottom confocal plane at original resolution with enlarged panels that were upscaled by bilinear filtering. See corresponding Figure 7 for more details.
